# Supplementary material for: In Silico Analysis of Temperature-Induced Structural, Stability, and Flexibility Modulations in Camel Cytochrome c
Source: Animals (Basel). 2025 Jan 28;15(3):381. doi: 10.3390/ani15030381 (PMC11815751; doi:10.3390/ani15030381)
Supplement: Supplementary file 1 [file animals-15-00381-s001.zip › animals-3392705-supplementary.pdf]

Supplementary

Table S1. Parameters used for MD simulation during NVT, NPT and MD production run. Here, the parameter ref_t is 245 K, 280 K, 303 K, 308 K, and 320 K, for each of the case.

NVT	Cut-off	NPT	Cut-off	MD run	Cut-off
nsteps	50000	nsteps	500000	nsteps	50000000
dt	0.002	dt	0.002	dt	0.002
nstenergy	500	nstenergy	500	nstxout	0
nstlog	500	nstlog	500	nstvout	0
nstxout-compressed	500	nstxout-compressed	500	nstxtcout	1000
lincs_iter	1	lincs_iter	1	nstenergy	1000
lincs_order	4	lincs_order	4	nstlog	1000
nstlist	20	nstlist	20	lincs_iter	1
rlist	1.2	rlist	1.2	lincs_order	4
rvdw-switch	1	rvdw-switch	1	nstlist	5
rvdw	1.2	rvdw	1.2	rlist	1.2
rcoulomb	1.2	rcoulomb	1.2	rlistlong	1.2
pme_order	4	pme_order	4	rvdw-switch	1
fourierspacing	0.16	fourierspacing	0.16	rcoulomb	1.2
tau_t	0.1	tau_t	0.1	rvdw	1.2
ref_t	X (specific for each case)	ref_t	X (specific for each case)	pme_order	4
gen_temp	310	tau_p	2	fourierspacing	0.16
gen_seed	-1	ref_p	1	tau_t	0.1
		compressibility	4.50e-05	ref_t	X (specific for each case)
				tau_p	4
				ref_p	1
				compressibility	4.50e-05
